# CD4^+^CD25^-^Foxp3^+^ T cells: a marker for lupus nephritis?

**DOI:** 10.1186/ar4553

**Published:** 2014-04-28

**Authors:** Michael Bonelli, Lisa Göschl, Stephan Blüml, Thomas Karonitsch, Carl-Walter Steiner, Günter Steiner, Josef S Smolen, Clemens Scheinecker

**Affiliations:** 1 Division of Rheumatology, Internal Medicine III, General Hospital of Vienna, Medical University of Vienna (MUW), Waehringer Guertel 18-20, Vienna A-1090, Austria

## Abstract

**Introduction:**

Systemic lupus erythematosus (SLE) is a heterogenous autoimmune disease, which can affect different organs. Increased proportions of CD4^+^CD25^-^Foxp3^+^ T cells have been described in SLE patients. The exact role of this cell population in SLE patients still remains unclear. We therefore analyzed this T cell subset in a large cohort of SLE patients with different organ manifestations.

**Methods:**

Phenotypic analyses, proportions and absolute cell numbers of CD4^+^CD25^-^Foxp3^+^ T cells were determined by flow cytometry (FACS) in healthy controls (HC) (n = 36) and SLE patients (n = 61) with different organ manifestations. CD4^+^CD25^-^Foxp3^+^ T cells were correlated with clinical data, the immunosuppressive therapy and different disease activity indices. In patients with active glomerulonephritis, CD4^+^CD25^-^Foxp3^+^ T cells were analyzed in urine sediment samples. Time course analyses of CD4^+^CD25^-^Foxp3^+^ T cells were performed in patients with active disease activity before and after treatment with cyclophosphamide and prednisone.

**Results:**

CD4^+^CD25^-^Foxp3^+^ T cells were significantly increased in active SLE patients and the majority expressed Helios. Detailed analysis of this patient cohort revealed increased proportions of CD4^+^CD25^-^Foxp3^+^ T cells in SLE patients with renal involvement. CD4^+^CD25^-^Foxp3^+^ T cells were also detected in urine sediment samples of patients with active glomerulonephritis and correlated with the extent of proteinuria.

**Conclusion:**

CD4^+^CD25^-^Foxp3^+^ T cells resemble regulatory rather than activated T cells. Comparative analysis of CD4^+^CD25^-^Foxp3^+^ T cells in SLE patients revealed a significant association of this newly described cell population with active nephritis. Therefore CD4^+^CD25^-^Foxp3^+^ T cells might serve as an important tool to recognize and monitor SLE patients with renal involvement.

## Introduction

Regulatory T cells (Treg) constitute on average 1 to 2% of human peripheral blood mononuclear cells (PBMC) and are characterized by their capacity to actively suppress T cell proliferation *in vitro*[1]. Treg play an important role in T cell homeostasis and are critical regulators of immune tolerance. Quantitative and/or qualitative deficiencies of Treg have been suggested to contribute to the development of autoimmune diseases [2-8].

Treg are best characterized by high expression levels of the IL-2 receptor α-chain (CD25) [9,10]. In addition the forkhead family transcription factor (Foxp3) has been described as a highly specific intracellular marker molecule for Treg [11-14]. Helios, a member of the Ikaros transcription factor family, has recently been described as a specific marker for thymic-derived Foxp3+ regulatory T cells [15,16]. In addition it has been shown that proportions but not absolute numbers of Foxp3^+^Helios^+^ T cell are increased in systemic lupus erythematosus (SLE) patients and that Foxp3^+^Helios^-^ T cells are capable of effector cytokine production [17].

We have recently identified a novel subset of CD4^+^Foxp3^+^ Treg that does not express CD25 surface molecules (CD4^+^CD25^-^Foxp3^+^) [18]. CD4^+^CD25^-^Foxp3^+^ share phenotypic characteristics with conventional CD4^+^CD25^+^Foxp3^+^ Treg and convey lower, but still considerable suppression of T cell proliferation, though not of IFN-γ production, *in vitro*. Increased proportions of CD4^+^CD25^-^Foxp3^+^ Treg are observed in particular in SLE patients, a finding that has widely been confirmed [19-23]. However, the origin, the precise functional role and the potential pathogenetic involvement of CD4^+^CD25^-^Foxp3^+^ Treg are still enigmatic.

In this study we therefore evaluated if the presence of CD4^+^CD25^-^Foxp3^+^ cells is associated with a particular phenotype of organ manifestations in SLE patients. Our data reveal that CD4^+^CD25^-^Foxp3^+^ that share phenotypic characteristics with regulatory T cells are increased in patients with lupus nephritis.

## Materials and methods

### Patients and controls

SLE patients (n = 61; mean age 45 ± 16.8 years) who fulfilled at least four of the revised SLE criteria of the American College of Rheumatology [24] were randomly selected from our outpatient clinic. Healthy volunteers served as a healthy control (HC) population (n = 36; mean age 42 ± 15.7 years). The disease activity of SLE patients was assessed using the SLE disease activity index (SLEDAI) [25], the European Consensus Lupus Activity Measurement (ECLAM) score [26] and the SLE index score (SIS) [27]. A detailed patient characteristic is shown in Table 1. Patients were divided into two groups according to their disease activity as determined by the SLEDAI score. A SLEDAI score ≥6 was defined as high disease activity and a SLEDAI score of <6 as low disease activity. Depending on their clinical status we divided the patients into groups with active and no active organ involvement at the time of the patient’s blood draw. Active organ involvement was defined by clinical and laboratory parameters. The following items were considered to be indicative for active organ involvement: active skin involvement defined as lupus rash, discoid lupus or photosensitivity; active joint involvement defined as arthritis with synovial swelling; active hematologic involvement defined as thrombocytopenia, lymphocytopenia, or leukocytopenia or (Coombs test positive) hemolytic anemia; active renal involvement defined as nephritis with proteinuria >0.5 g protein/24 h and/or active nephritic sediment. In all patients with renal involvement a renal biopsy had been performed and glomerulonephritis was classified according to the World Health Organization (WHO) classification [28]. All patients with renal manifestation were classified as having glomerulonephritis WHO III or IV.

**Table 1:** Demographic and clinical characteristics of systemic lupus erythematosus patients

**Characteristic**	**Value**
Age, years, mean ± SD	40.4 ± 14.5
Gender, female/male, n (%)	56 (91.8%)/5 (8.2%)
Disease duration years, mean ± SD	5.4 ± 4.9
Anti-ds DNA Ab-positive, n (%)	23 (62.3%)
C3, mean ± SD	90.5 ± 26.6
C4, mean ± SD	14.3 ± 7.1
CH50, mean ± SD	110.6 ± 31.9
SLEDAI, mean ± SD	4.2 ± 4.2
ECLAM, mean ± SD	1.9 ± 1.9
SIS, mean ± SD	3.6 ± 2.9
Current prednisone dose, mg/day, mean ± SD	10.4 ± 15.3
Concurrent immunosuppressive therapy, n (%)	
Hydroxychloroquine	22 (36.1%)
Mycophenolate mofetil	9 (14.8%)
Cyclophosphamide	6 (9.8%)
Azathioprine	8 (13.1%)
Methotrexate	5 (8.2%)
Organ involvement, n (%)	
Skin/active	37 (60.7.1%)/8 (13.1%)
Hematologic/active	23 (37.7%)/10 (16.4%)
Arthritis/active	22 (36.1%)/3 (4.9%)
Serositis/active	8 (13.1%)/2 (3.3%)
Renal/active	25 (40.9%)/13 (21.3%)

SLEDAI, systemic lupus erythematosus disease activity index; ECLAM, European Consensus Lupus Activity Measurement; SIS, systemic lupus erythematosus index score.

Ethical approval for this study was granted by the local ethics committee of the Medical University of Vienna and internal review board of the Medical University of Vienna, Austria. Patients gave written informed consent to participate in the study and agreed that the findings of the study will be published in a scientific journal.

### Antibodies

The following mAb/conjugates were used in this study: R-phycoerythrin (PE), phycoerythrin-cyanin5 (PE-Cy5) and allophycocyanin (APC), PE-Cy7-conjugated mAb against CD4 (SK3) and CD25 (2A3) were purchased from Becton Dickinson (San Jose, CA, USA); mAb against Foxp3 (236A/E7) was obtained from eBiosciences (San Diego, CA, USA); mAb against CD14 (RMO52) was obtained from Beckman Coulter (Fullerton, CA, USA), mAb against Helios (22 F6) was obtained from Biolegend (San Diego, CA, USA).

### Phenotypic analyses

PBMC were isolated from heparinized blood by layering over LSM 1077 Lymphocyte Separation Medium (PAA laboratories, Pasching, Austria) and density gradient centrifugation at 400 × g. PBMC were resuspended in PBS/3% human Ig (Baxter International Inc., Vienna Austria) in order to block Fc receptors and prevent non-specific antibody binding, incubated for 15 minutes at 4°C in the dark and stained with different combinations of fluorescein isothiocyanate (FITC), PE, PE-Cy5, APC and PE-Cy7 and APC-Cy7-conjugated mAb and their appropriate isotype controls. Intracellular staining for Foxp3 was performed according to the instructions of the manufacturer eBiosciences (San Diego, CA, USA). The samples were analyzed on a FACSCanto II (Becton Dickinson Immunocytometry Systems, San Jose, CA, USA) using FACSDiva software v.6.1.2 (BD Bioscience) and FlowJo software v 7.1.2 (Tree Star). Lymphocytes were gated according to the Forward Scatter and Side Scatter. In addition gated CD4^+^ cells were analyzed for the expression of CD25 and Foxp3. Proportions of CD25^+^Foxp3^+^ and CD25^-^Foxp3^+^ cells within the gated CD4^+^ cells are shown. Absolute numbers of cells were calculated from whole blood counts obtained from routine laboratory testing.

### Urine analysis

In patients with renal involvement, the extent of proteinuria was determined by measuring total protein in a 24-h urine collection specimen. Cells from 24-h urine samples from three patients were isolated by density gradient centrifugation. Cells were subjected to phenotypic analysis as described above.

### Statistical analysis

Values are shown throughout the manuscript as mean ± standard error of the mean (SEM), unless stated otherwise. Proportions of lymphocyte subpopulations were compared using the Student *t*-test for normally distributed populations and if the variables were not normally distributed we used the Kruskal-Wallis test. Relationships between separate groups of data were examined using the Pearson correlation coefficient and Spearman rank correlation test. A *P*-value equal or less than 0.05 was considered significant in all statistical tests. All statistical analyses were performed using GraphPad Prism (Graph Pad Prism 4.0 by Graph Pad software Inc.) and SPSS (SPSS 12.0 by SPSS software Inc.).

## Results

### Increased proportions of CD4^+^CD25^-^Foxp3^+^ T cells in patients with active SLE

**Figure 1:**
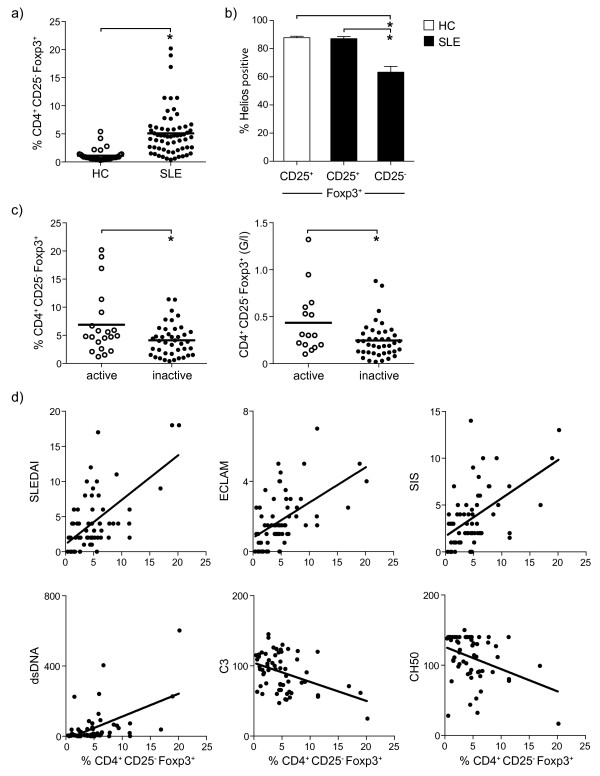
**Increased proportions of CD4**^**+**^**CD25**^**-**^**Foxp3**^**+ **^**T cells in patients with active systemic lupus erythematosus (SLE).** Proportions of CD4^+^CD25^-^Foxp3^+^ T cells were analyzed by fluorescence-activated cell sorting (FACS) in SLE patients and healthy controls (HC). **(a)** A significant increase in the percentage of CD4^+^CD25^-^Foxp3^+^ T cells was observed in SLE patients (n = 61) as compared to HC (n = 36) (*P* <0.0001). **(b)** The proportion of Helios + cells was significantly reduced in CD4^+^CD25^-^Foxp3^+^ T cells from SLE patients as compared to CD4^+^CD25^+^Foxp3^+^ T cells from HC (*P* <0.0001) and SLE patients (*P* <0.0001). **(c)** Percentage (*P* = 0.01) as well as absolute numbers (*P* = 0.039) of CD4^+^CD25^-^Foxp3^+^ T cells were significantly increased in patients with active SLE as compared to patients with inactive SLE. **(d)** A significant correlation was observed for the percentage of CD4^+^CD25^-^Foxp3^+^ T cells and the SLE disease activity index (SLEDAI) (*r* = 0.5; *P* <0.001), European Consensus Lupus Activity Measurement (ECLAM) (*r* = 0.54; *P* <0.001) and SLE index score (SIS) score (*r* = 0.5; *P* <0.001), as well as the levels of dsDNA antibodies (*r* = 0.4; *P* <0.001), C3 (*r* = 0.3; *P* = 0.01) and CH50 (*r* = 0.3; *P* = 0.01). *Significant differences.

Freshly isolated PBMC from HC (n = 36) and SLE patients (n = 61) were analyzed by fluorescence-activated cell sorting (FACS) for proportions of CD4^+^CD25^-^Foxp3^+^ T cells. As shown in Figure 1a proportions of CD4^+^CD25^-^Foxp3^+^ T cells were significantly increased in patients with SLE (5.1 ± 0.5%) as compared to HC (1.1 ± 0.2%) (*P* <0.0001). To further investigate their origin we compared CD4^+^Foxp3^+^CD25^+/-T^ cells from HC and SLE patients for the expression of Helios, as a marker for thymic origin [15,16]. As shown in Figure 1b, similar proportions of CD4^+^CD25^+^Foxp3^+^ T cells from HC (87.8 ± 1%) and SLE patients (87.1 ± 1.5%) expressed Helios. On the other hand we observed a significantly decreased percentage of Helios^+^ cells in CD4^+^CD25^-^Foxp3^+^ T cells in SLE patients (63.3 ± 4%) as compared to CD4^+^Foxp3^+^CD25^+^ from HC (*P* <0.0001) or SLE patients (*P* <0.0001). In addition proportions of CD4^+^ CD25^-^Foxp3^+^ T cells showed a significant correlation with CD4^+^CD25^+^Foxp3^+^ (*r* = 0.7; *P* <0.0001) but not with CD4^+^CD25^+^Foxp3^-^ (*r* = 0.17; *P* <0.32) in SLE patients (Additional file 1: Figure S1). This together with our previous results [18] suggests that the majority of CD4^+^CD25^-^Foxp3^+^ resemble thymic derived Treg.

To determine whether the increase in CD4^+^CD25^-^Foxp3^+^ T cells in SLE patients is linked to a higher clinical disease activity, SLE patients were divided into patients with active (SLEDAI score ≥6) and inactive (SLEDAI score <6) disease (Figure 1c). Significantly higher proportions of CD4^+^CD25^-^Foxp3^+^ T cells were observed in active (6.9 ± 1.3%) as compared to inactive (4.1 ± 0.5%) SLE (*P* = 0.01). A similar result was observed when we compared absolute numbers of CD4^+^CD25^-^Foxp3^+^ T cells in active and inactive SLE (*P* = 0.039).

We further correlated CD4^+^CD25^-^Foxp3^+^ cells with levels of disease activity using established scores, and found significant correlations with all tested scores: SLEDAI (*r* = 0.5; *P* <0.001), ECLAM (*r* = 0.54; *P* <0.001) and SIS (*r* = 0.5; *P* <0.001). In addition we observed a significant correlation with anti-dsDNA antibody levels (*r* = 0.4; *P* <0.001) and a significant inverse correlation between the levels of complement factor C3 (*r* = 0.3; *P* = 0.01) and CH50 (*r* = 0.3; *P* = 0.01) and proportions of CD4^+^CD25^-^Foxp3^+^ T cells (Figure 1d). These correlations were also significant for absolute cell numbers of CD4^+^CD25^-^Foxp3^+^ T cells (data not shown). In summary CD4^+^CD25^-^Foxp3^+^ T cells showed features of regulatory T cells and were clearly linked to patients with higher disease activity.

### Increased proportions of CD4^+^CD25^-^Foxp3^+^ T cells are observed in SLE patients treated with cyclophosphamide

**Figure 2:**
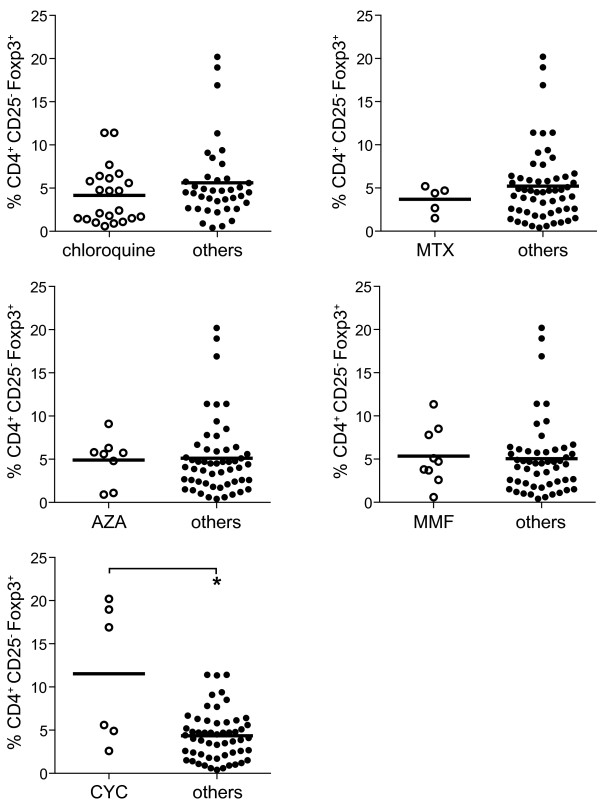
**Increased proportions of CD4**^**+**^**CD25**^**-**^**Foxp3**^**+ **^**T cells in patients treated with cyclophosphamide.** Patients were subdivided into groups according to their immunosuppressive treatment. MMF, mycofenolate mofetil; MTX, methotrexate; AZA, azathioprine; CYC, cycophosphamide. Significantly higher proportions of CD4^+^CD25^-^Foxp3^+^ T cells were observed in systemic lupus erythematosus (SLE) patients who were treated with cyclophosphamide as compared to patients with other treatment regimes (*P* = 0.0001). *Significant differences.

Next we determined whether increased proportions of CD4^+^CD25^-^Foxp3^+^ T cells are associated with a specific immunosuppressive treatment regimen. As depicted in Figure 2, significantly higher proportions of CD4^+^CD25^-^Foxp3^+^ T cells were observed in SLE patients who were treated with cyclophosphamide (11.5 ± 0.3%) as compared to patients with other treatment regimes (4.3 ± 0.4%; *P* = 0.0001). No differences in the proportions of CD4^+^CD25^-^Foxp3^+^ T cells were observed in SLE patients treated with chloroquine, methotrexate, azathioprine or mycophenolate mofetil. A more detailed analysis of the SLE patients treated with cyclophosphamide, revealed that all patients suffered from nephritis. However, when we compared absolute cell numbers of CD4^+^CD25^-^Foxp3^+^ T cells no significant differences were observed between the different treatment regimens (data not shown).

### Prednisone treatment does not influence CD4^+^CD25^-^Foxp3^+^ T cells

**Figure 3:**
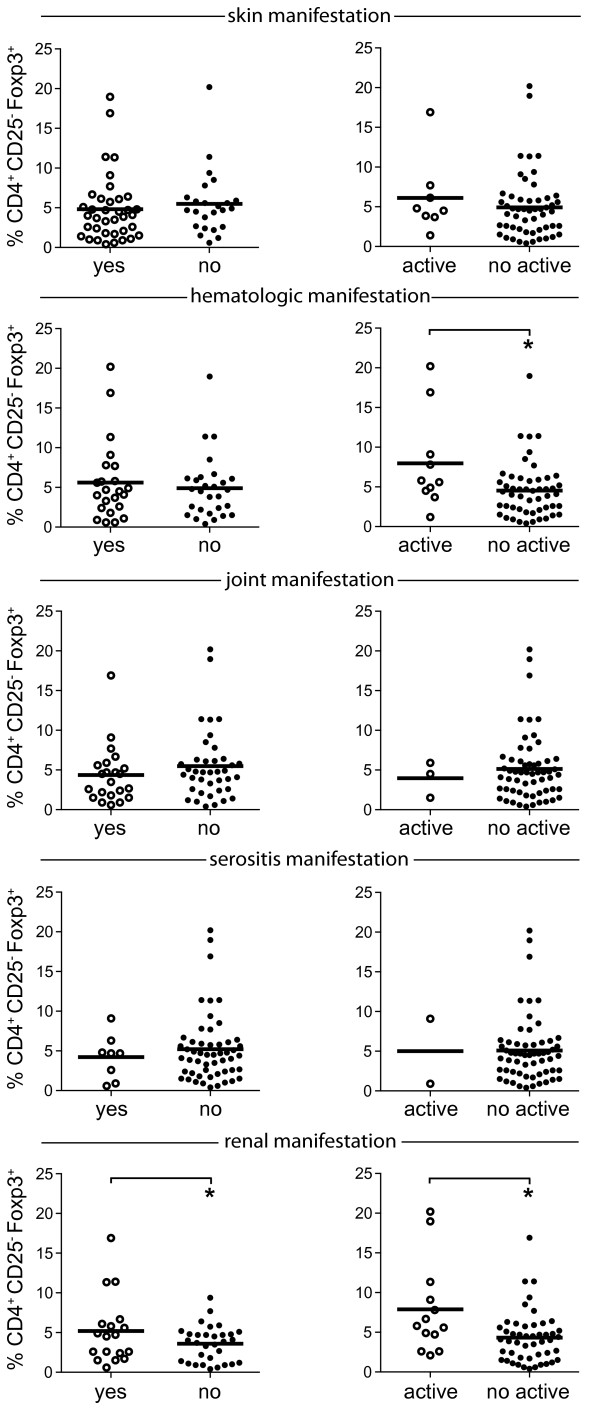
**Increased proportions of CD4**^**+**^**CD25**^**-**^**Foxp3**^**+ **^**T cells in patients with renal manifestation.** Systemic lupus erythematosus (SLE) patients were divided into different groups according to their organ manifestations. In addition patients were subdivided into groups with (active) and without (no active) organ involvement and at the time of the patient’s blood draw. A significant increase in proportions of CD4^+^CD25^-^Foxp3^+^ T cells was observed in patients with renal manifestation (*P* = 0.005) and in patients with active nephritis (*P* = 0.005). Increased proportions of CD4^+^CD25^-^Foxp3^+^ T cells were also observed in patients with active hematologic manifestations as compared to patients with no active hematologic manifestations (*P* = 0.02). *Significant differences.

Significant correlation was observed for proportions (*r* = 0.3; *P* = 0.02) and absolute cell numbers (*r* = 0.3; *P* = 0.02) of CD4^+^CD25^-^Foxp3^+^ T cells with the daily prednisone dose (Figure 3a). As patients with active SLE are usually treated with higher glucocorticoid doses, we correlated the daily dose of prednisone with the SLEDAI score. Indeed we observed a significant correlation between the daily prednisone dose and the SLEDAI score (*r* = 0.5; *P* = 0.0003; Figure 3a), suggesting that increased proportions of CD4^+^CD25^-^Foxp3^+^ T cells in SLE patients treated with a higher glucocorticoid dose can be explained by higher disease activity. Longitudinal analysis of a patient with high disease activity, who was treated with different doses of prednisone, confirmed that prednisone did not substantially affect proportions of CD4^+^CD25^-^Foxp3^+^ T cells (Figure 3b).

### Increased proportions of CD4^+^CD25^-^Foxp3^+^ T cells in SLE patients with renal involvement

**Figure 4:**
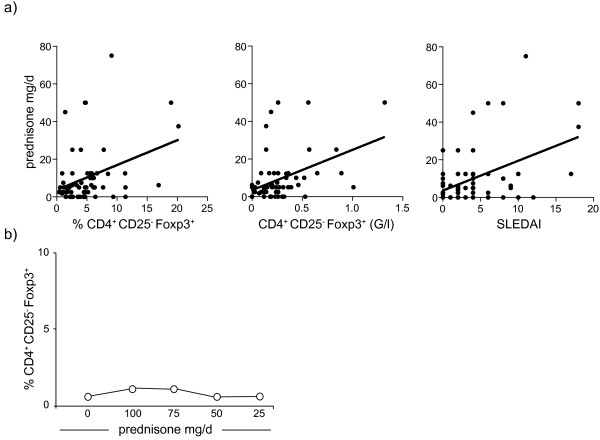
**The daily glucocorticoid dose correlates with% CD4**^**+**^**CD25**^**-**^**Foxp3**^**+ **^**T cells and the systemic lupus erythematosus disease activity index (SLEDAI) disease activity score. (a)** The daily glucocorticoid dose of systemic lupus erythematosus (SLE) patients was significantly correlated with the percentage of CD4^+^CD25^-^Foxp3^+^ T cells (*r* = 0.3; *P* = 0.02), and the absolute cell numbers of CD4^+^CD25^-^Foxp3^+^ T cells (*r* = 0.3; *P* = 0.02), as well as the SLEDAI score (*r* = 0.5; *P* = 0.0003). **(b)** Longitudinal analysis of the percentage of CD4^+^CD25^-^Foxp3^+^ T cells is shown in a patient with high disease activity treated with prednisone.

SLE represents a heterogenous disease with a variety of different organ manifestations. Until now, an association of specific organ manifestation with proportions of CD4^+^CD25^-^Foxp3^+^ T cells has not been investigated. We therefore divided all SLE patients into different groups according to their organ manifestation. In addition we further subdivided all patients into groups with active and no active organ involvement at the time of Treg assessment (Figure 4).

We observed a significant increase in proportions of CD4^+^CD25^-^Foxp3^+^ T cells in patients with renal involvement (6.7 ± 1.1%) as compared to patients with no renal involvement (3.6 ± 0.4%; *P* = 0.005) and in patients with active nephritis (7.9 ± 1.6%) as compared to SLE patients with no active nephritis (4.3 ± 0.5%; *P* = 0.005). In addition, increased proportions of CD4^+^CD25^-^Foxp3^+^ T cells were also observed in patients with active hematologic involvement (7.9 ± 1.9%) as compared to patients with no active hematologic involvement (4.5 ± 0.5%; *P* = 0.02). However, when we excluded patients with active nephritis from the group of patients with active hematologic involvement, the difference lost its significance, suggesting that the observed difference in patients with active hematologic involvement was due to their concomitant renal involvement (data not shown). No differences in proportions of CD4^+^CD25^-^Foxp3^+^ T cells were observed for patients with other organ manifestations (Figure 4). When we compared absolute cell numbers of CD4^+^CD25^-^Foxp3^+^ T cells in patients with different organ manifestations a significant difference was only observed in patients with renal involvement (Additional file 2: Figure S2).

In summary, the observed increase in the percentage and absolute cell numbers of CD4^+^CD25^-^Foxp3^+^ T cells in patients with renal involvement suggests that CD4^+^CD25^-^Foxp3^+^ T cells are associated with this particular organ manifestation.

### CD4^+^CD25^-^Foxp3^+^ T cells can be detected in urine samples of SLE patients with renal involvement and correlate with the extent of proteinuria

**Figure 5:**
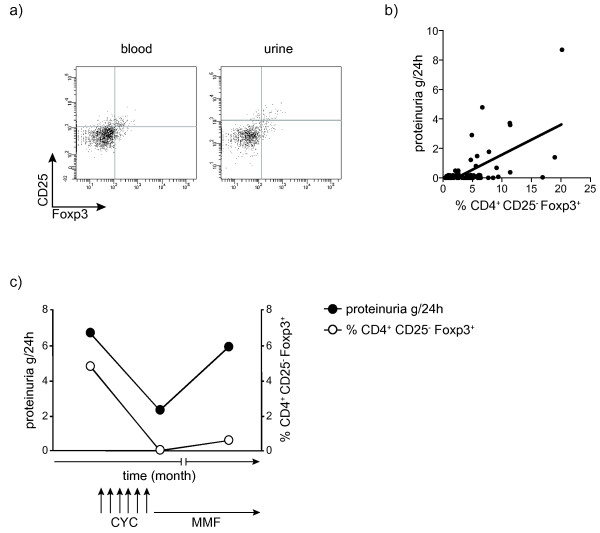
**CD4**^**+**^**CD25**^**-**^**Foxp3**^**+ **^**T cells can be detected in urine samples of patients with active nephritis and correlate with the extent of proteinuria. (a)** CD4^+^CD25^-^Foxp3^+^ T cells were analyzed by fluorescence-activated cell sorting (FACS) in blood and urine sediment samples from SLE patients with active nephritis. One representative dot plot is shown. **(b)** The percentage of CD4^+^CD25^-^Foxp3^+^ T cells was correlated with the extent of proteinuria (*r* = 0.4; *P* = 0.002). **(c)** One representative longitudinal analysis of the percentage of CD4^+^CD25^-^Foxp3^+^ T cells together with the extent of proteinuria is shown in a patient with newly diagnosed active glomerulonephritis treated with a monthly boli of cyclophosphamide. CYC, cyclophosphamide; MMF, mycofenolate mofetil.

Next we analyzed CD4^+^CD25^-^Foxp3^+^ T cells in patients with nephritis in more detail. As depicted in Figure 5a, CD4^+^CD25^-^Foxp3^+^ T cells were also detectable in the urine sediment of a patient with lupus nephritis. Proportions of CD4^+^CD25^-^Foxp3^+^ T cells in the urine sediment were comparable with proportions of CD4^+^CD25^-^Foxp3^+^ T cells in the peripheral blood. Moreover we observed a significant correlation between proportions of CD4^+^CD25^-^Foxp3^+^ T cells and the extent of proteinuria (*r* = 0.4; *P* = 0.002) in patients with renal involvement (Figure 5b).

In order to analyze whether the correlation of CD4^+^CD25^-^Foxp3^+^ T cells with the extent of proteinuria is linked to a treatment response, we performed longitudinal analysis in three SLE patients with newly diagnosed active kidney disease who were treated with cyclophosphamide. As can be seen from one example in Figure 5c we observed a decrease in proportions of CD4^+^CD25^-^Foxp3^+^ T cells after the initiation of therapy with cyclophosphamide. This was paralleled by a decline in the extent of proteinuria. After seven boli of cyclophosphamide the treatment was continued with mycophenolat mofetil. Under this treatment, an increase in proportions of CD4^+^CD25^-^Foxp3^+^ T cells together with an increase in the extent of proteinuria was observed.

## Discussion

In this study we analyzed the recently identified population of CD4^+^CD25^-^Foxp3^+^ T cells in a cohort of SLE patients. Extended phenotypic analysis based on the expression of the Ikaros transcription factor family member Helios support our previous assumption [18] that the majority of CD4^+^CD25^-^Foxp3^+^ T cells resemble Treg. We further described a connection between the disease activity and peripheral blood CD4^+^CD25^-^Foxp3^+^ T cells. In addition we showed for the first time that the increment of CD4^+^CD25^-^Foxp3^+^ T cells is linked to a specific organ manifestation, namely to renal involvement. A correlation between CD4^+^CD25^-^Foxp3^+^ cells and the extent of proteinuria further support the idea of CD4^+^CD25^-^Foxp3^+^ cells as a marker to recognize and monitor patients with renal involvement.

Initial studies regarding frequencies of Treg in the peripheral blood of SLE patients have generated controversial results. Most groups, including our own, have described decreased proportions of CD4^+^CD25^high^ Treg in SLE patients as compared to healthy controls and observed an inverse correlation of Treg numbers with clinical disease activity [7,19,29-31]. However, other studies reported unaltered proportions or even increased proportions of Treg in SLE patients and a positive correlation with disease activity [17,19,32-34].

Among the overall population of CD4^+^CD25^+^ Treg we have recently identified a population of CD4^+^Foxp3^+^ T cells that lack the expression of CD25. Proportions of CD4^+^CD25^-^Foxp3^+^ T cells were on average five-fold higher in SLE patients as compared to HC [18]. Meanwhile this finding has been confirmed by several other groups who also described increased proportions of CD4^+^Foxp3^+^ T cells that are CD25-negative or express only low levels of CD25 in SLE patients [19,21,23,34].

Helios has recently been identified as a marker to distinguish thymic-derived Treg from Treg that are induced in the periphery [15,16]. Within this study we observed lower proportions of Helios^+^ cells among CD4^+^CD25^-^Foxp3^+^ T cells as compared to CD4^+^CD25^+^Foxp3^+^ T cells, suggesting that CD4^+^CD25^-^Foxp3^+^ T cells might reflect a combination of thymic-derived and peripherally induced Treg cells. In addition we found significant correlation between CD4^+^CD25^-^Foxp3^+^ T cells and CD4^+^CD25^+^Foxp3^+^ bona fide Treg cells, but not with CD4^+^CD25^+^Foxp3^-^ T cells, representing activated T cells. This together suggests that CD4^+^CD25^-^Foxp3^+^ T cells represent regulatory T cells rather then activated T cells.

Concerning the influence of the disease activity on proportions of CD4^+^CD25^-^Foxp3^+^ T cells, however, certain discrepancies exist. We therefore addressed this question in more detail in an extended cohort of SLE patients. Thereby we were able to describe a correlation between proportions of CD4^+^CD25^-^Foxp3^+^ T cells and disease activity in SLE patients using three different disease activity scores. Moreover, we observed significant correlation between CD4^+^CD25^-^Foxp3^+^ T cells with levels of anti-dsDNA antibodies and complement levels. In line with our data Lin *et al*. reported a significant increase of CD4^+^Foxp3^+^ T cells in patients with active SLE, determined by the SLEDAI score, as compared to patients with inactive SLE or HC. In contrast, however, they observed no correlation between CD4^+^CD25^-^Foxp3^+^ T cells and disease activity, the complement levels and concentrations of anti-dsDNA antibodies [34]. Suen *et al*. also described increased proportions of CD25^low^Treg and CD25^-^Treg in patients with active and inactive SLE as compared to HC. Although they focused mainly on the ratio between different Treg subsets and effector T cells, they observed increased proportions of CD25^-^Treg in patients with low complement levels. Similar observations of increased numbers of CD4^+^CD25^-^Foxp3^+^ T cells in patients with active as compared to inactive SLE or HC were further made by Zhang *et al*. Moreover they also reported direct correlation between proportions of CD4^+^CD25^-^Foxp3^+^ T cells and concentrations of antibodies against dsDNA, whereas no correlation was observed for the C3 levels and the SLEDAI score [19]. These discrepancies might be on the one hand explained by different definitions of patients with active and inactive SLE. On the other hand differences might exist in the composition of the study populations, in particular in regard to different organ manifestation as discussed in more detail below.

To further exclude a treatment effect on the expression of CD25 on T cells we compared proportions of CD4^+^CD25^-^Foxp3^+^ T cells in SLE patients with different treatment regimens. Thereby we only observed increased proportions in patients treated with cyclophosphamide. Further analysis, however, revealed that all cyclophosphamide-treated patients also suffered from active nephritis. In addition we observed no significant differences for absolute cell numbers of CD4^+^CD25^-^Foxp3^+^ T cells in SLE patients with other treatment regimens.

Significant correlation was found between CD4^+^CD25^-^Foxp3^+^ T cells and the daily cortisone dose. Patients with active SLE, however, are usually treated with a higher cortisone dose and in fact this was confirmed by a significant correlation between the daily cortisone dose and the SLEDAI score. In addition we were able to follow one newly diagnosed SLE patient with high disease activity, and skin-, blood- and joint-manifestation, who was treated with prednisone. This resulted in gradually reduced disease activity without a concomitant substantial effect on proportions of CD4^+^CD25^-^Foxp3^+^ T cells. Likewise, also other studies did not describe a correlation between Treg and the daily cortisone dose in SLE patients [17,23] except for Zhang *et al*. who observed decreased proportions of CD4^+^CD25^-^Foxp3^+^ T cells after treatment of active SLE with cortisone [19]. An explanation for this discrepancy might be the different treatment regimen, as all patients in the study by Zhang *et al*. also received cyclophosphamide at the same time. On the other hand, differences in the organ manifestations might contribute to this discrepancy.

As a systemic autoimmune disease SLE can affect almost any organ system and most patients display multi-organ involvement with unpredictable exacerbations and remissions with protean clinical manifestations. The most common organs that are involved are the skin, the musculoskeletal system and the kidneys. Organ systems may be involved singly or in any combination. Until now the association of a certain organ manifestation with the presence of different T cell subsets has not been investigated so far. As we also observed an increase of CD4^+^CD25^-^Foxp3^+^ T cells in cyclophosphamide-treated patients who suffered from active nephritis we compared CD4^+^CD25^-^Foxp3^+^ T cells in patients with active and no active organ involvement. Interestingly, patients with renal involvement, especially patients with active nephritis, displayed increased proportions of CD4^+^CD25^-^Foxp3^+^ T cells, suggesting that the observed increase was mainly associated with renal organ involvement. We also observed elevated numbers in patients with active blood involvement. This observation, however, was mainly due to an overlap in the organ manifestation since most of the patients with hematologic manifestations also suffered from renal involvement. When we compared absolute cell numbers of CD4^+^CD25^-^Foxp3^+^ T cells, we only observed a significant difference in patients with renal involvement, but not in patients with hematologic manifestation. Furthermore CD4^+^CD25^-^Foxp3^+^ T cells were also detected in the urine sediment of patients with active nephritis. In addition we also observed significant correlation between the CD4^+^CD25^-^Foxp3^+^ T cells and the extent of proteinuria, reflecting the disease activity in patients with active nephritis. Longitudinal analysis of patients with active nephritis revealed a decline in CD4^+^CD25^-^Foxp3^+^ T cells under cyclophosphamide treatment in parallel to a drop in the extent of proteinuria and the disease activity. The parallel decrease and increase of CD4^+^CD25^-^Foxp3^+^ T cells with the disease activity in SLE patients with renal manifestation might reflect the potential of CD4^+^CD25^-^Foxp3^+^ T cells as a biomarker to diagnose and monitor SLE patients with active kidney involvement.

One of the key questions remains whether CD4^+^CD25^-^Foxp3^+^ T cells actually represent a beneficial counter-mechanism against autoimmunity, or on the contrary, if these cells are part of the damaging auto-immunological machinery of SLE. In fact Treg display a certain plasticity, in particular under inflammatory conditions, and might even transdifferentiate into pathogenic effector T cells. However, until an activation-independent surface marker molecule is found for Treg, this question cannot be answered to date. On the other hand, irrespective of their functional capacity, CD4^+^CD25^-^Foxp3^+^ T cells might be of interest in SLE patients as a marker of disease activity, organ manifestation or treatment response.

In conclusion we were able to demonstrate that this newly described cell population of CD4^+^CD25^-^Foxp3^+^ T cells is clearly influenced by the disease activity and that an increase of this cell population is mainly observed in patients with renal involvement. The correlation with the extent of proteinuria suggests that the assessment of CD4^+^CD25^-^Foxp3^+^ cells can be used as a tool to recognize and monitor SLE patients with renal involvement.

## Conclusion

CD4^+^CD25^-^Foxp3^+^ T cells resemble a marker for lupus nephritis. CD4^+^CD25^-^Foxp3^+^ T cells represent a subpopulation of Treg and are increased in SLE patients with high disease activity. Here we show that CD4^+^CD25^-^Foxp3^+^ T cells are significantly increased in patients with renal involvement. Longitudinal data of patients with lupus nephritis further support the idea that CD4^+^CD25^-^Foxp3^+^ T cells might serve to recognize and monitor SLE patients with renal involvement.

## Abbreviations

APC: allophycocyanin; ECLAM: European Consensus Lupus Activity Measurement; FACS: fluorescence-activated cell sorting; Foxp3: forkhead family transcription factor; IFN: interferon; PBMC: peripheral blood mononuclear cells; PE: phycoerythrin; PE-Cy5: phycoerythrin-cyanin5; SEM: standard error of the mean; SIS: systemic lupus erythematosus index score; SLE: systemic lupus erythematosus; SLEDAI: systemic lupus erythematosus disease activity index; Treg: regulatory T cells.

## Competing interests

The authors declare that they have no competing interests.

## Authors’ contributions

MB: conception and design, data collection and analysis, manuscript writing, critical revision and final approval of the manuscript. LG: data collection and analysis, critical revision and final approval of the manuscript. SB: data collection and analysis, critical revision and final approval of the manuscript. TK: data collection and analysis, critical revision and final approval of the manuscript. CWS: data collection and analysis, critical revision and final approval of the manuscript. GS: conception and design, financial support, critical revision and final approval of the manuscript. JSS: conception and design, manuscript writing, financial support, critical revision and final approval of the manuscript. CS: conception and design, manuscript writing, critical revision and final approval of the manuscript. All authors read and approved the final manuscript.

## Supplementary Material

Additional file 1: Figure S1 CD4^+^CD25^-^Foxp3^+^ T cells correlate with regulatory T cells but not with activated T cells. CD4^+^CD25^-^Foxp3^+^ T cells from systemic lupus erythematosus (SLE) patients showed a significant correlation with CD4^+^CD25^+^Foxp3^+^ (*r* = 0.7; *P* <0.0001) but not with CD4^+^ CD25^+^Foxp3^-^ (*r* = 0.17; *P* = 0.32). Click here for file

Additional file 2: Figure S2 Increased absolute cell numbers of CD4^+^CD25^-^Foxp3^+^ T cells in patients with renal manifestation. Systemic lupus erythematosus (SLE) patients were divided into different groups according to their organ manifestations. In addition patients were subdivided into groups with active and no active organ involvement. A significant increase in absolute cell numbers of CD4^+^CD25^-^Foxp3^+^ T cells was observed in patients with renal involvement (*P* = 0.01) and in patients with active nephritis (*P* = 0.04). *Significant differences. Click here for file
