# Supplementary figures and images for: CD4+CD25-Foxp3+ T cells: a marker for lupus nephritis?
Source: Arthritis Res Ther. 2014 Apr 28;16(2):R104. doi: 10.1186/ar4553 (PMC4060257; doi:10.1186/ar4553)

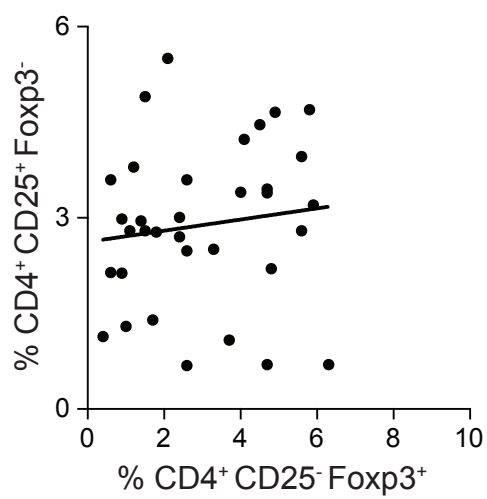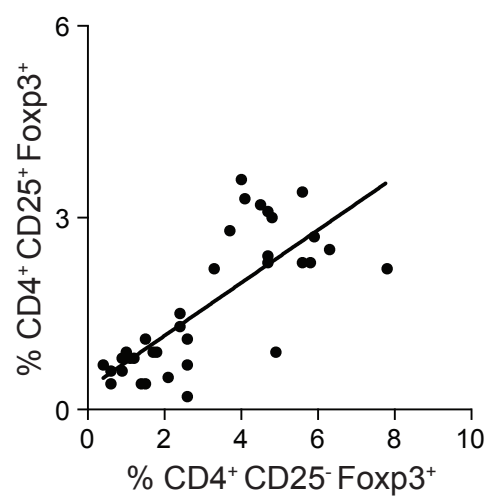

Supplement: Additional file 1: Figure S1 — CD4+CD25-Foxp3+ T cells correlate with regulatory T cells but not with activated T cells. CD4+CD25-Foxp3+ T cells from systemic lupus erythematosus (SLE) patients showed a significant correlation with CD4+CD25+Foxp3+ (r = 0.7; P <0.0001) but not with CD4+ CD25+Foxp3- (r = 0.17; P = 0.32). [file ar4553-S1.pdf]

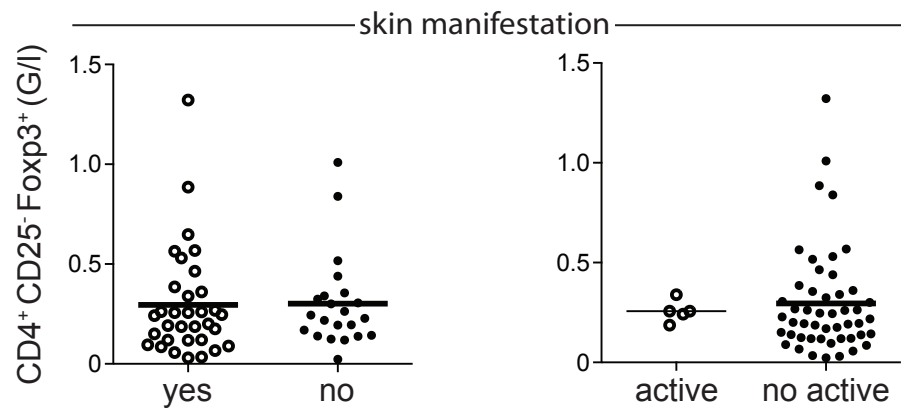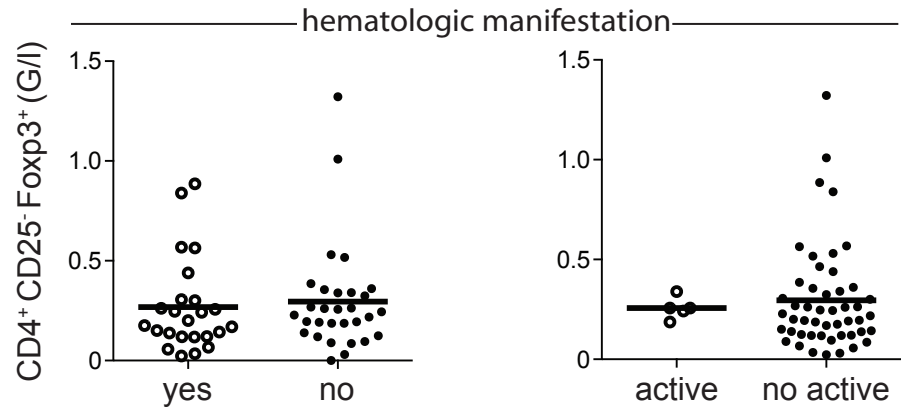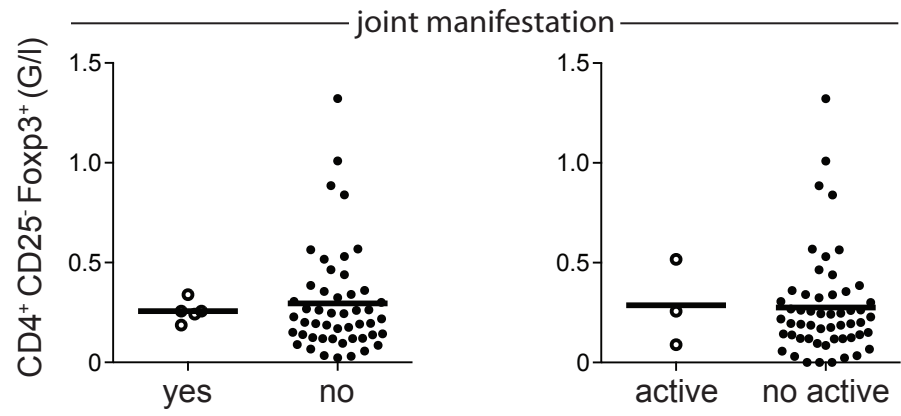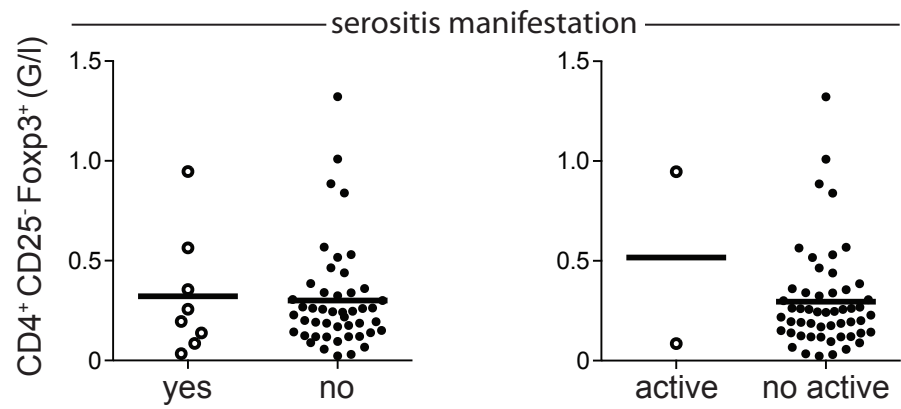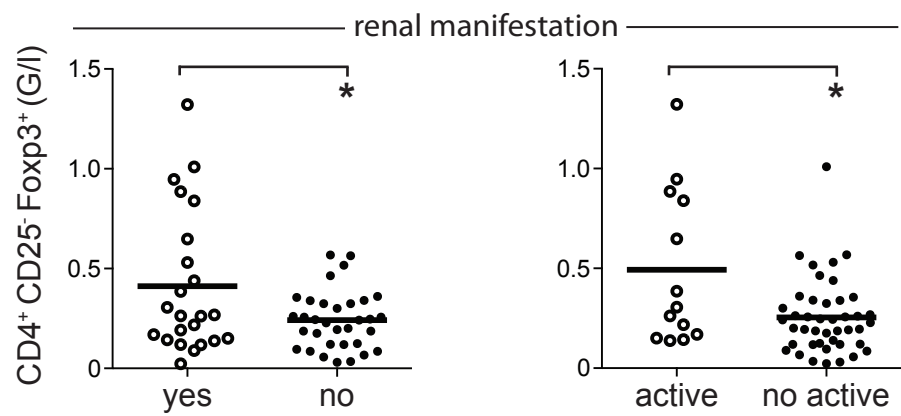

Supplement: Additional file 2: Figure S2 — Increased absolute cell numbers of CD4+CD25-Foxp3+ T cells in patients with renal manifestation. Systemic lupus erythematosus (SLE) patients were divided into different groups according to their organ manifestations. In addition patients were subdivided into groups with active and no active organ involvement. A significant increase in absolute cell numbers of CD4+CD25-Foxp3+ T cells was observed in patients with renal involvement (P = 0.01) and in patients with active nephritis (P = 0.04). *Significant differences. [file ar4553-S2.pdf]
